# Association between activities related to routes of infection and clinical manifestations of melioidosis

**DOI:** 10.1016/j.cmi.2015.09.016

**Published:** 2016-01

**Authors:** C. Lim, S.J. Peacock, D. Limmathurotsakul

**Affiliations:** 1) Mahidol-Oxford Tropical Medicine Research Unit, Faculty of Tropical Medicine, Mahidol University, Bangkok, Thailand; 2) Department of Medicine, University of Cambridge, Addenbrooke’s Hospital, Cambridge, UK; 3) London School of Hygiene and Tropical Medicine, London, UK; 4) Department of Tropical Hygiene, Faculty of Tropical Medicine, Mahidol University, Bangkok, Thailand

**Keywords:** Bacteraemia, *Burkholderia pseudomallei*, ingestion, inhalation, inoculation, melioidosis, pneumonia

## Abstract

We sought associations between route of infection by *Burkholderia pseudomallei* and clinical manifestations in 330 cases of melioidosis in northeast Thailand using bivariate multivariable logistic regression models. Activities related to skin inoculation were negatively associated with bacteraemia, activities related to ingestion were associated with bacteraemia, and activities related to inhalation were associated with pneumonia. Our study suggests that route of infection is one of the factors related to clinical manifestations of melioidosis.

Melioidosis is an infectious disease caused by *Burkholderia pseudomallei* [1], which is highly endemic in South East Asia and northern Australia where the organism is commonly found in soil and ground water [2]. The most common risk factor for melioidosis is diabetes mellitus, which is present in around half of cases [1]. Infection occurs via skin inoculation, ingestion or inhalation of the organism [3]. Clinical manifestations of melioidosis are highly variable and range from localized skin infection to acute septicaemia and/or pneumonia, with or without abscesses in internal organs [1], [4]. The basis for such variability is largely unknown, but one factor may be the route by which the infecting inoculum is acquired [4]. Several studies in animal models have described an association between routes of *B. pseudomallei* challenge and clinical manifestations [5], [6], [7]. This is also supported by circumstantial evidence relating to the proportion of patients with melioidosis who present with pneumonia following severe weather events, which has been proposed to relate to bacterial inhalation [8]. However, *B. pseudomallei* is rarely isolated from the air even during typhoons [2], [9], and patients with a known inoculation event can develop pneumonia as a result of bacterial dissemination. Also, the clinical manifestations of melioidosis associated with acquisition via ingestion have not been evaluated. Here, we explore the association between routes of infection and clinical manifestations of melioidosis.

Data on 330 cases of culture-proven melioidosis were drawn from a previous study of the activities of daily living associated with the onset of melioidosis, which was conducted at Sappasithiprasong Hospital, Ubon Ratchathani, northeast Thailand between July 2010 and December 2011 [3]. Each case was interviewed and information was collected on specified activities of daily living during the 30 days preceding the onset of symptoms using a standardized study form. Relatives were interviewed if patients were not capable of answering questions. The study was approved by the research ethics committees of Sappasithiprasong Hospital, and the Faculty of Tropical Medicine, Mahidol University. Written informed consent was obtained from all participants [3]. Age, gender, diabetes, activities reported previously to be associated with a risk of melioidosis [3], and onset of symptoms during the rainy season were included in the analysis. The rainy season was defined as the period from June to November. Activities were grouped into those related to skin inoculation, ingestion or inhalation, as described previously [3]. The clinical manifestations evaluated were bacteraemia and pneumonia. Bacteraemia was defined as blood culture positive for *B. pseudomallei* at any time during hospital admission. Pneumonia was defined as the presence of clinical features plus radiographic changes consistent with this diagnosis, and/or sputum culture positive for *B. pseudomallei*, at any time during hospital admission. The association between route of infection and hepatosplenic abscess was not evaluated because only patients who survived until the culture results were available underwent abdominal ultrasound investigation.

Bivariate multivariable logistic regression models were used to evaluate the association between activities related to routes of infection and clinical manifestations. As the definitive route of infection cannot be determined in natural infection, we used activities of daily living as independent factors from which we inferred the routes of infection [3]. Clinical manifestations were the outcome of interest. Univariable and multivariable regression models were used to explore associations between activities and each clinical manifestation. A bivariate multivariable logistic regression model was then used to evaluate independent associations between activities and the two clinical manifestations because patients can present with both manifestations at the same time. The final model was developed using purposeful selection in which all activities associated with a risk of melioidosis acquisition [3], and variables independently associated with clinical manifestations were included. The association between the two clinical manifestations was explored using Kendall’s tau-b. All analyses were performed using Stata13.0 (StataCorp, College Station, TX, USA), except the bivariate analyses were performed using R (Version 0.98.1091).

A total of 330 culture-confirmed melioidosis cases were included in the analysis. The median age was 53 years (interquartile range 45–63 years), 212 (64%) were men, 151 (46%) had diabetes and 119 (36%) died. Overall, 177 (54%) patients had bacteraemia, and 166 (50%) had pneumonia. A total of 79 (24%) patients had bacteraemic pneumonia, and a weak negative association between bacteraemia and pneumonia was observed (Kendall’s tau-b, −0.12; 95% CI −0.05 to −0.19, p 0.03). Other common clinical manifestations were hepatosplenic abscess (*n* = 58, 18%), skin and soft tissue infection (*n* = 59, 18%), genitourinary tract infection (*n* = 55, 17%) and septic arthritis (*n* = 20, 6%) (see Supplementary material, Table S1).

Using univariable and multivariable logistic regression models, we found that age, diabetes mellitus and presentation to hospital during the rainy season were not associated with bacteraemia or pneumonia (see Supplementary material, Tables S2, S3, S4 and S5). We then used bivariate multivariable logistic regression to evaluate the combination of independent associations between activities at risk versus bacteraemia and/or pneumonia (Table 1). We found that working in a rice field, undertaking other activities involving exposure to soil or water, and being male were independently and negatively associated with bacteraemia. Patients who worked in rice fields had a 70% lower odds of having bacteraemia (adjusted odds ratio (aOR) 0.3, 95% CI 0.1–0.6), whereas males had a 40% lower odds of having bacteraemia (aOR 0.6, 95% CI 0.3–1.0). In contrast, patients who drank untreated water had 120% higher odds of having bacteraemia (aOR 2.2, 95% CI 1.1–4.2). Patients with a history of water inhalation (aOR 1.8, 95% CI 1.0–3.2) and those who smoked (aOR 1.8, 95% CI 1.0–3.0) were independently associated with having pneumonia.

Our study suggests that infection via skin exposure is negatively associated with bacteraemia and that ingestion is associated with bacteraemia. Our findings are consistent with those from the experimental melioidosis mouse model, in which bacterial dissemination occurred more rapidly in mice given an oral *B. pseudomallei* challenge compared with those infected via the subcutaneous route [7]. An association between activities related to ingestion and bacteraemia is also supported by a recent report of two patients with melioidosis linked to drinking *B. pseudomallei*-contaminated water, who developed bacteraemia [10]. The negative association between activities related to skin exposure and bacteraemia is also supported by the observation that patients with melioidosis who present with skin infection in Australia rarely have bacteraemia [11], [12]. We also evaluated the specific association between activities related to inoculation and clinical manifestations with skin and subcutaneous infection. No statistically significant associations were observed (all p values >0.20).

The association between smoking and pneumonic melioidosis is consistent with a previous study in Australia [13]. Smoking decreases the effectiveness of the local immune response and increases the risk of infection. The association between water inhalation and pneumonia is consistent with previous reports in tsunami survivors and in patients with a history of near-drowning [14]. These findings support the hypothesis that inhalation of *B. pseudomallei* is associated with pneumonia. We did not find an association between outdoor exposure to rain and pneumonia. There is evidence that mice infected with a high inoculum of *B. pseudomallei* via the subcutaneous route also develop systemic infection [5], [7], and based on this it is possible that a proportion of patients presenting with pneumonia and bacteraemia were infected via skin exposure with a high inoculating dose. The association between ingestion and hepatosplenic and parotid abscesses has been discussed [4], but our data set could not be used to evaluate this association.

Our study suggests that route of infection is one of the factors related to clinical manifestations of melioidosis. However, none of the activities related to routes of infection could be used to predict clinical manifestations with a high level of confidence. Further studies are required to evaluate bacterial and human genetic factors associated with clinical manifestations of melioidosis.

## Funding

The study was funded by the Wellcome Trust [090219/Z09/Z]. DL is supported by a Wellcome Trust Intermediate Fellowship [101103/Z/13/Z], and CL is supported by a Wellcome Trust Masters fellowship [100484/Z/12/Z].

## Transparency declaration

All authors have reported that there are no conflicts.

## Figures and Tables

**Table 1:** Association between activities relevant to route of bacterial acquisition and the development of bacteraemia and/or pneumonia

	Adjusted OR for bacteraemia^a^ (95% CI)	p	Adjusted OR for pneumonia^a^ (95% CI)	p
Activities related to skin exposure				
No activities involving exposure to soil or water	1.0	0.004	1.0	0.13
Working in a rice field	0.3 (0.1–0.6)		1.3 (0.7–2.6)	
Other activities involving exposure to soil or water	0.4 (0.2–1.1)		2.4 (1.0–5.6)	
Open wound	1.4 (0.8–2.5)	0.23	1.0 (0.6–1.8)	0.95
Activities related to ingestion				
Eating food contaminated with soil or dust	1.3 (0.8–2.1)	0.34	1.0 (0.6–1.5)	0.85
Drinking untreated water	2.2 (1.1–4.2)	0.02	1.1 (0.6–2.1)	0.72
Activities related to inhalation				
Outdoor exposure to dust cloud	1.0 (0.6–1.6)	0.91	0.9 (0.5–1.4)	0.60
Outdoor exposure to rain	0.8 (0.5–1.3)	0.30	1.1 (0.7–1.9)	0.57
History of water inhalation^b^	0.9 (0.5–1.6)	0.72	1.8 (1.0–3.2)	0.04
Other risk factors				
Current smoker	1.1 (0.7–1.9)	0.66	1.8 (1.0–3.0)	0.04
Taking oral steroids	0.9 (0.4–2.3)	0.89	2.0 (0.8–4.8)	0.13
Male gender	0.6 (0.3–1.0)	0.04	0.8 (0.5–1.3)	0.37

a Determined using bivariate multivariable logistic regression model. Four cases (1%) were excluded from the model because they did not answer questions about eating food contaminated with soil or dust (*n* = 3), or about steroid intake (*n* = 1).

b Having a coughing fit while drinking water or accidental choking when diving under water.
