# Supplementary material for: Association between activities related to routes of infection and clinical manifestations of melioidosis
Source: Clin Microbiol Infect. 2016 Jan;22(1):79.e1–3. doi: 10.1016/j.cmi.2015.09.016 (PMC4721533; doi:10.1016/j.cmi.2015.09.016)
Supplement: Supplementary file 1 [file mmc1.docx]

**Supplementary Table 1.** Clinical manifestation of 330 culture-proven melioidosis patients

| Clinical manifestation ^a^ | All patients  (N=330) | Patients with bacteremia and pneumonia  (N=79) | Patients with bacteremia and without pneumonia  (N=98) | Patients with pneumonia and without bacteraemia (N=87) | Patients without bacteraemia and pneumonia  (N=66) |
| --- | --- | --- | --- | --- | --- |
| Bacteremia | 177 (54%) | 79 (100%) | 98 (100%) | 0 (0%) | 0 (0%) |
| Pneumonia | 166 (50%) | 79 (100%) | 0 (0%) | 87 (100%) | 0 (0%) |
| Skin and subcutaneous infection | 59 (18%) | 4 (5%) | 15 (15%) | 10 (12%) | 30 (45%) |
| Urinary tract infection | 52 (16%) | 21 (27%) | 13 (13%) | 7 (8%) | 11 (17%) |
| Liver abscesses | 34 (10%) | 7 (9%) | 13 (13%) | 5 (6%) | 9 (14%) |
| Splenic abscesses | 43 (13%) | 8 (10%) | 18 (18%) | 9 (10%) | 8 (12%) |
| Arthritis | 20 (6%) | 3 (4%) | 11 (11%) | 3 (3%) | 3 (5%) |
| Prostatic infection | 5 (2%) | 1 (1%) | 1 (1%) | 0 (0%) | 3 (5%) |
| Parotitis | 5 (2%) | 1 (1%) | 1 (1%) | 0 (0%) | 3 (5%) |
| Other organs ^b^ | 8 (2%) | 0 (0%) | 0 (0%) | 1 (1%) | 7 (11%) |

^a^ a patient could have more than one clinical manifestation

^b^ pleuritis (n=4), pericarditis (n=2), lymphadenitis (n=1) and paravertebral abscess (n=1)

**Supplementary Table 2.** Association between activities relevant to route of bacterial acquisition and the development of bacteremia

|  | No. of bacteremia / Total No. (%) | Crude odds ratio (95% CI) ^a^ | P value |
| --- | --- | --- | --- |
| Activities related to skin inoculation |  |  |  |
| No activities involving exposure to soil or water | 39/53 (73.6) | 1.0 | 0.004 |
| Working in a rice field | 114/234 (48.7) | 0.3 (0.2-0.7) |  |
| Other activities involving exposure to soil or water | 24/43 (55.8) | 0.5 (0.2-1.1) |  |
| No open wound | 138/261 (52.9) | 1.0 | 0.59 |
| Open wound | 39/69 (56.5) | 1.2 (0.7-2.0) |  |
| Activities related to ingestion |  |  |  |
| Not eating food contaminated with soil or dust | 103/195 (52.8) ^b^ | 1.0 | 0.76 |
| Eating food contaminated with soil or dust | 72/132 (54.6) ^b^ | 1.1 (0.7-1.7) |  |
| Not drinking untreated water | 22/53 (41.5) | 1.0 | 0.05 |
| Drinking untreated water | 155/277 (56.0) | 1.8 (1.0-3.2) |  |
| Activities related to inhalation |  |  |  |
| No outdoor exposure to dust cloud | 69/125 (55.2) | 1.0 | 0.66 |
| Outdoor exposure to dust cloud | 108/205 (52.7) | 0.9 (0.6-1.4) |  |
| No outdoor exposure to rain | 95/163 (58.3) | 1.0 | 0.10 |
| Outdoor exposure to rain | 82/167 (49.1) | 0.7 (0.4-1.1) |  |
| No history of water inhalation | 141/261 (54.0) | 1.0 | 0.78 |
| History of water inhalation | 36/69 (52.2) | 0.9 (0.5-1.6) |  |
| Other risk factors |  |  |  |
| Not current smoker | 117/214 (54.7) | 1.0 | 0.61 |
| Current smoker | 60/116 (51.7) | 0.9 (0.6-1.4) |  |
| Not taking oral steroids | 160/302 (53.0) ^b^ | 1.0 | 0.53 |
| Taking oral steroids | 16/27 (59.3) ^b^ | 1.3 (0.6-2.9) |  |
| Female gender | 73/118 (61.9) | 1.0 | 0.03 |
| Male gender | 104/212 (49.1) | 0.6 (0.4-0.9) |  |
| No diabetes mellitus | 94/179 (52.5) | 1.0 | 0.66 |
| Diabetes mellitus | 83/151 (55.0) | 1.1 (0.7-1.7) |  |
| Age <40yrs | 23/45 (51.1) | 1.0 | 0.94 |
| Age 40-59yrs | 95/176 (54.0) | 1.1 (0.6-2.2) |  |
| Age ≥60yrs | 59/109 (54.1) | 1.1 (0.6-2.3) |  |
| Onset of symptoms during the non-rainy season | 42/87 (48.3) | 1.0 | 0.24 |
| Onset of symptoms during the rainy season ^c^ | 135/243 (55.6) | 1.3 (0.8-2.2) |  |

^a^ Determined using univariable logistic regression model

^b^ Four cases did not answer questions in the original study about eating food contaminated with soil or dust (n=3), or about steroid intake (n=1)

^c^ June to November

**Supplementary Table 3.** Adjusted association between activities relevant to route of bacterial acquisition and the development of bacteremia

|  | Adjusted Odds ratio (95% CI) ^a^ | P value |
| --- | --- | --- |
| Activities related to skin inoculation |  |  |
| No activities involving exposure to soil or water | 1.0 | 0.004 |
| Working in a rice field | 0.3 (0.1-0.6) |  |
| Other activities involving exposure to soil or water | 0.4 (0.2-1.1) |  |
| Open wound | 1.4 (0.8-2.5) | 0.23 |
| Activities related to ingestion |  |  |
| Eating food contamination with soil or dust | 1.3 (0.8-2.0) | 0.35 |
| Drinking untreated water | 2.2 (1.1-4.2) | 0.02 |
| Activities related to inhalation |  |  |
| Outdoor exposure to dust cloud | 1.0 (0.6-1.6) | 0.90 |
| Outdoor exposure to rain | 0.8 (0.5-1.2) | 0.29 |
| History of water inhalation | 0.9 (0.5-1.6) | 0.70 |
| Other risk factors |  |  |
| Current smoker | 1.1 (0.7-1.9) | 0.66 |
| Taking oral steroids | 0.9 (0.4-2.2) | 0.87 |
| Male gender | 0.6 (0.3-1.0) | 0.04 |

^a^ Determined using Multivariable logistic regression model. Four cases (1%) were excluded from the model because they did not answer questions about eating food contaminated with soil or dust (n=3), or about steroid intake (n=1).

**Supplementary Table 4.** Association between activities relevant to route of bacterial acquisition and the development of pneumonia

|  | No. of pneumonia / Total No. (%) | Crude odds ratio (95% CI) ^a^ | P value |
| --- | --- | --- | --- |
| Activities related to skin inoculation |  |  |  |
| No activities involving exposure to soil or water | 22/53 (41.5) | 1.0 | 0.11 |
| Working in a rice field | 117/234 (50.0) | 1.4 (0.8-2.6) |  |
| Other activities involving exposure to soil or water | 27/43 (62.8) | 2.4 (1.0-5.4) |  |
| No open wound | 129/261 (49.4) | 1.0 | 0.54 |
| Open wound | 37/69 (53.6) | 1.2 (0.7-2.0) |  |
| Activities related to ingestion |  |  |  |
| Not eating food contaminated with soil or dust | 96/195 (49.2) ^b^ | 1.0 | 0.79 |
| Eating food contamination with soil or dust | 67/132 (50.8) ^b^ | 1.1 (0.7-1.7) |  |
| Not drinking untreated water | 24/53 (45.3) | 1.0 | 0.43 |
| Drinking untreated water | 142/277 (51.3) | 1.3 (0.7-2.3) |  |
| Activities related to inhalation |  |  |  |
| No outdoor exposure to dust cloud | 63/125 (50.4) | 1.0 | 0.98 |
| Outdoor exposure to dust cloud | 103/205 (50.2) | 1.0 (0.6-1.6) |  |
| No outdoor exposure to rain | 77/163 (47.2) | 1.0 | 0.27 |
| Outdoor exposure to rain | 89/167 (53.3) | 1.3 (0.8-2.0) |  |
| No history of water inhalation | 123/261 (47.1) | 1.0 | 0.03 |
| History of water inhalation | 43/69 (62.3) | 1.9 (1.1-3.2) |  |
| Other risk factors |  |  |  |
| Not current smoker | 98/214 (45.8) | 1.0 | 0.03 |
| Current smoker | 68/116 (58.6) | 1.7 (1.1-2.6) |  |
| Not taking oral steroids | 148/302 (49.0) ^b^ | 1.0 | 0.08 |
| Taking oral steroids | 18/27 (66.7) ^b^ | 2.1 (0.9-4.8) |  |
| Female gender | 59/118 (50.0) | 1.0 | 0.94 |
| Male gender | 107/212 (50.5) | 1.0 (0.6-1.6) |  |
| No diabetes mellitus | 91/179 (50.8) | 1.0 | 0.83 |
| Diabetes mellitus | 75/151 (49.7) | 1.0 (0.6-1.5) |  |
| Age <40yrs | 20/45 (44.4) | 1.0 | 0.68 |
| Age 40-59yrs | 91/176 (51.7) | 1.3 (0.7-2.6) |  |
| Age ≥60yrs | 55/109 (50.5) | 1.3 (0.6-2.6) |  |
| Onset of symptoms during the non-rainy season | 43/87 (49.4) | 1.0 | 0.85 |
| Onset of symptoms during the rainy season ^c^ | 123/243 (50.6) | 1.0 (0.6-1.7) |  |

^a^ Determined using Univariable logistic regression model

^b^ Four cases did not answer questions in the original study about eating food contaminated with soil or dust (n=3), or about steroid intake (n=1)

^c^ June to November

**Supplementary Table 5.** Adjusted association between activities relevant to route of bacterial acquisition and the development of pneumonia

|  | Adjusted Odds ratio (95% CI) ^a^ | P value |
| --- | --- | --- |
| Activities related to skin inoculation |  |  |
| No activities involving exposure to soil or water | 1.0 | 0.13 |
| Working in a rice field | 1.3 (0.7-2.6) |  |
| Other activities involving exposure to soil or water | 2.3 (1.0-5.6) |  |
| Open wound | 1.0 (0.6-1.8) | 0.96 |
| Activities related to ingestion |  |  |
| Eating food contamination with soil or dust | 1.0 (0.6-1.5) | 0.85 |
| Drinking untreated water | 1.1 (0.6-2.1) | 0.71 |
| Activities related to inhalation |  |  |
| Outdoor exposure to dust cloud | 0.9 (0.5-1.4) | 0.60 |
| Outdoor exposure to rain | 1.2 (0.7-1.9) | 0.56 |
| History of water inhalation | 1.8 (1.0-3.2) | 0.04 |
| Other risk factors |  |  |
| Current smoker | 1.8 (1.0-3.0) | 0.04 |
| Taking oral steroids | 2.0 (0.8-4.7) | 0.13 |
| Male gender | 0.8 (0.5-1.3) | 0.37 |

^a^ Determined using Multivariable logistic regression model. Four cases were excluded from the model because they did not answer questions in about eating food contaminated with soil or dust (n=3), or about steroid intake (n=1).
